# Experiences and Perceptions Around Eating Among Older Adults With Serious Mental Illness

**DOI:** 10.1093/geroni/igaf122.2431

**Published:** 2025-12-31

**Authors:** Erin Hubbard, Heather Leutwyler

**Affiliations:** University of California, San Francisco, San Francisco, California, United States; University of California, San Francisco, San Francisco, California, United States

## Abstract

**Background:**

People with serious mental illness (SMI) have, on average, a shortened life expectancy of about 25 years compared to those without SMI. They experience an increased level of comorbidities such as metabolic syndrome (comprised of abdominal obesity, hypertension, impaired glucose tolerance, and hypertriglyceridemia), thus contributing to type 2 diabetes and cardiovascular disease. External circumstances such as precarious living environments and poor socioeconomic status can influence access to healthy food, and psychiatric symptoms may further impede healthy eating habits. To better understand facilitators and barriers to healthy eating among community-dwelling older adults with SMI, we report the results of a qualitative analysis.

**Methods:**

We interviewed 7 community-dwelling older adults with SMI (mean age 64.57, SD 6.705, range 59-76). A qualitative analysis using constructed grounded theory methodology was conducted to assess participants’ experiences and perceptions around eating and access to food.

**Results:**

Two main themes emerged in our analyses: 1. Early life influence on eating habits and 2. Convenience. Participants indicated that the early home environment can positively or negatively influence eating habits throughout life. Cooking skills and eating habits were often acquired early in life. Participants also described how convenience guided the types of food they consume. This convenience approach to nutrition often determined whether participants were eating non-nutrient-dense food. Participants expressed interest in learning about cooking or nutrition.

**Conclusion:**

Mental health programs that incorporate cooking experiences and nutrition or health groups provide important opportunities for people to learn and practice changes to diet and eating habits.

